# Kidney transplantation in low- and middle-income countries: the Transplant Links experience

**DOI:** 10.1007/s00467-023-06129-z

**Published:** 2023-09-02

**Authors:** Lydia E. Roberts, Amrit Kaur, Jennie Jewitt-Harris, Andrew Ready, David V. Milford

**Affiliations:** 1 University Hospitals of Leicester, Leicester, UK; 2 College of Life Sciences, University of Leicester, Leicester, UK; 3 Royal Manchester Children’s Hospital, Manchester, UK; 4 Transplant Links Community, Camberley, UK

**Keywords:** Paediatric kidney transplantation, Low- and middle-income countries, Kidney transplant mentoring, Challenges, Barriers

## Abstract

Paediatric kidney failure is a global problem responsible for significant childhood morbidity and mortality. The gold-standard treatment is kidney transplantation. However, the availability of kidney transplantation remains limited in some low- and middle-income countries (LMICs). Transplant Links Community (TLC) is a UK-based charity that mentors units in LMICs wishing to start kidney transplantation; the ultimate goal is for these units to become self-sufficient. TLC provides this support through in-person training visits and skill transfer, plus direct mentorship from the UK that is maintained over many years. From such mentoring programmes, it is evident that there are numerous challenges in the initial establishment and long-term maintenance of kidney transplant services, with specific and unique barriers applying to setting up paediatric transplant programmes compared to their adult counterparts. This review summarises TLC’s first-hand experience of developing paediatric kidney transplantation services in LMICs over the past 15 years, the challenges encountered, and the major ongoing barriers that must be addressed to facilitate further progress in delivering transplantation services to children globally.

## Introduction

Paediatric kidney failure is a global problem often described as a hidden epidemic. A kidney transplant is acknowledged as the gold-standard treatment for patients with kidney failure, associated with the best patient outcomes [1] and greater cost-effectiveness when compared to dialysis [2]. Due to a lack of national kidney registries in low- and middle-income countries (LMICs), the true burden of morbidity and mortality from paediatric chronic kidney disease (CKD) and kidney failure, as well as an accurate assessment of the availability of and access to kidney replacement therapy (KRT) and specialist paediatric nephrology care, is unknown [3, 4]. However, reports from tertiary centres in LMICs do reveal a notable CKD prevalence [5–9], although such estimates are likely to underestimate actual numbers significantly. Despite this clear clinical need, several studies highlight the current widespread deficit in providing paediatric KRT [10–14]. This is in stark contrast to most high-income countries, where the treatment of kidney failure is a streamlined process in which almost no patient dies for lack of treatment [15]. Furthermore, while supporting the development of kidney transplantation in LMICs, the non-governmental organisation (NGO) Transplant Links has encountered many complex barriers to both the initiation and achievement of sustainability of paediatric kidney transplant programmes.

## Transplant links community

Transplant Links Community (TLC) was founded in 2006 and is a UK-registered charitable organisation (NGO), independent of other societies [16]. The charity’s aim is to mentor and train kidney units in LMICs wishing to initiate or further develop kidney transplantation programmes. It was set up by British doctors working in transplantation with an altruistic desire to share specialist skills and thereby improve the lives of patients with kidney failure in LMICs. TLC has a small management team and a medical faculty of over 50 NHS specialists (surgeons, nephrologists, nurses, and operating theatre technicians) who have commited to donating their time to this cause over a number of years.

Through its unique approach, TLC enables skill transfer in the performance of living donor transplantation, with the objective that transplant programmes can become self-sustaining. TLC works with partner-country multidisciplinary transplant teams, providing hands-on surgical mentoring, lectures, and training seminars at the partner units and in the form of online planning and MDT meetings, videos, podcasts, and online symposia. Continuous long-term support and partnerships are maintained to develop the infrastructure required for a kidney transplant programme. From the outset, TLC encourages the inclusion of children with CKD and the involvement of paediatricians when developing kidney transplant services.

Over the past 15 years, TLC has worked with transplant units in many countries across Africa, Asia, and the Caribbean [17]. Against the background of TLC’s broader activity, this article summarises TLC’s first-hand experience of developing paediatric kidney transplantation services in several LMICs during this period. The challenges to development identified during this experience are described and compared with the more widely reviewed challenges faced by the global transplant community.

## The TLC approach

### Transplantation

**Fig. 1:**
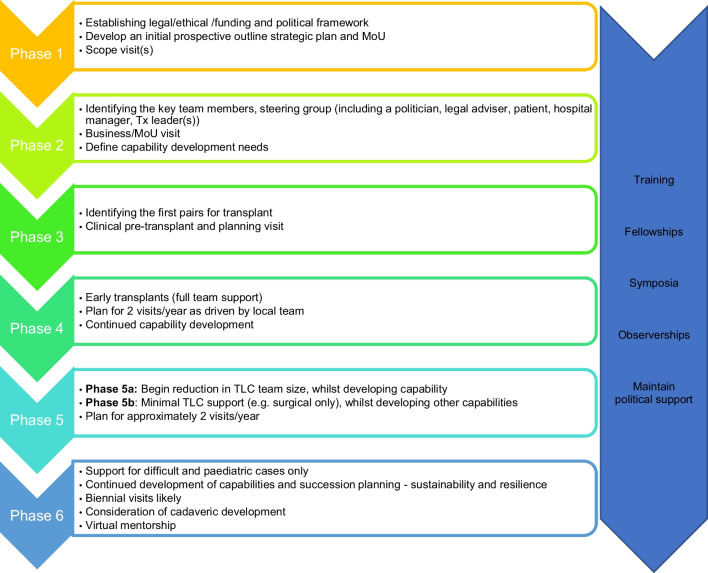
The six phases of development in the TLC model. MoU - memorandum of understanding; Tx - transplant

Recognising the commonly faced obstacles in establishing kidney transplant programmes, TLC has developed an innovative model that supports units in LMICs as they progress through the stages of development (Fig. 1). In most instances, local clinicians who recognise the clinical needs will contact TLC, having heard of its developments in other countries. These motivated champions of local patients with CKD then liaise with TLC to establish whether their centre is likely to be able to initiate and maintain a kidney transplant programme. Scoping visits to potential units by TLC are fundamental to this process. Such visits include the following:

- Assessing the experiential base of existing nephrology, surgical, and nursing personnel regarding kidney transplantation.
- Reviewing the appropriateness of medical facilities, including clinical areas, anaesthetic rooms, and operating theatres; identifying radiology and laboratory resources, including vascular imaging, tissue typing, and drug level monitoring.
- Supporting the local team in their interactions to obtain senior managerial and political support for their programme.

Paediatric kidney transplant services are invariably lacking in the early stages of unit development and must be set up de novo. To this end, TLC encourages the development of paediatric transplantation alongside adult transplant programmes. The availability of experienced paediatric nephrologists is a frequently encountered challenge; it is rare for units to have a resident paediatric nephrologist. However, some centres have started paediatric programmes with older paediatric patients who are faring poorly on dialysis and who may be managed by adult nephrologists as they progress toward a parent-donated live donor kidney transplant.

Once initial assessments are complete, central to TLC’s approach is ensuring local skills in kidney transplantation are developed and sustained. This is initially achieved by providing support and logistical guidance to establish the necessary infrastructure. After that, in-person TLC visits occur alongside frequent remote mentoring meetings online to assist local clinicians in arranging and then performing paediatric kidney transplants. TLC relies on a group of NHS specialists in transplantation (surgeons, nephrologists, nurses, and operating theatre technicians) who donate their time to travel to partner countries to share their skills in kidney transplantation and to provide significant remote support in the work-up, postoperative, and long-term phases.

Remote, Internet-based teaching and frequent clinical discussions complement such visits. In addition, opportunities are provided for multidisciplinary transplant team members to undertake learning visits to high-volume kidney transplant centres in the UK, involving a mixture of hands-on experience and observerships for both nurses and doctors.

Mentorship then becomes continuous, open-ended, and tailored to the specific needs of each partner centre until a point of sustainability is achieved. The more specialist nature, and lower caseload of paediatric kidney transplantation for individual units, mean such services are more complex to establish than their adult equivalents, with self-sufficiency being a greater challenge. The absence of paediatric nephrologists means care of the children falls to adult nephrologists. In one instance, TLC has facilitated the development of a consultant-led paediatric nephrology service by supporting the training of a paediatric nephrologist through a 2-year hands-on training programme at a UK tertiary nephrology centre, before taking up the first paediatric nephrologist consultant post in their home nation. Such specialist training takes considerable planning, resources, and identification of a suitable placement at an appropriate time for the trainee. It is therefore important that partner centres are aware of these factors and are committed from the outset to a long-term vision of paediatric transplantation development.

A bespoke approach is taken with each partner centre to overcome hurdles specific to their country, to match local needs, and to address skill transfer requirements. Each new transplant programme moves through six phases of progress, as outlined in Fig. 1.

The overall goal is the establishment of sustainable transplant programmes to ensure patients can continue to benefit in the future, with TLC acting as the catalyst but not the effector of progress. TLC does not measure the success of its partner centres by the number of transplants performed each year but rather on the progression through the development phases outlined above. Partnerships with TLC are long-term, with ongoing support throughout all developmental stages. One TLC partner unit has now progressed to phase 6; this progression took over 10 years to achieve. Such progress depends on teamwork, communication, and managerial and government support. Also, there must be fundraising to cover the costs of TLC support, teaching, visits, and, crucially, the generosity of NHS volunteers using their vacation time to travel and share skills.

### Patient and public involvement

As well as working closely with the selected centres, TLC strives to support local colleagues impacting their local kidney healthcare landscape. TLC has helped partner colleagues increase public and patient awareness of the benefits of kidney transplantation over other forms of KRT. This has included recruiting vital political and managerial support for kidney transplant programmes and helping to create demand for transplant programmes at other sites. Since children with CKD may often not progress to dialysis in LMICs, kidney centres have the challenges of establishing KRT services and potentially identifying children, not on dialysis, who would benefit from preemptive transplantation. Increasing awareness in patients and families, as well as politicians, healthcare managers, and hospital staff, of the benefits of kidney transplantation is vital in helping to facilitate treatment for those children in or approaching kidney failure [18]. Looking to the future, TLC is actively exploring the establishment of networks between partner centres so that new transplant units can ultimately act as referral centres for countries with too small a population to establish a transplant centre.

TLC has also worked with local clinicians to engage with politicians and policymakers, advocating for financial investment to ensure that programmes mentored by TLC are maintained, trained staff are retained, and teams are expanded as demand increases. TLC supports such lobbying in partner countries through assisting with the preparation of policy and strategy documents and attendance at stakeholder meetings, working alongside key members of the local clinical teams.

The achievements of centres mentored by TLC have helped prove that the blocks to developing successful paediatric transplantation services can be overcome, even though this may take time. Indeed, the model adopted by TLC has been shown to work when there is a long-term commitment from all stakeholders. However, despite frequent contact and continued channels of communication with politicians, TLC’s experience has frequently been that such long-term commitment from policymakers is very difficult to obtain. Paediatric kidney transplantation remains low on the political agenda in many of the countries in which TLC has worked, emphasising the absolute need for ongoing and persistent lobbying of politicians and policymakers.

## Paediatric kidney transplants undertaken in partnership with TLC

Over the past 15 years, TLC has assisted three transplant units in the Caribbean to perform adult and paediatric kidney transplants. We have decided not to name the specific units in this article so that the publication of their own data and case series, which TLC strongly encourages and supports, is not hindered. The centres do, however, provide representative examples of what could be expected in other LMICs. The following section is thus not intended to be an in-depth analysis of patient outcomes, but instead a review of the TLC experience of developing paediatric transplantation programmes at these centres.

Prior to writing this review, partner centres were contacted directly and asked to provide data for paediatric transplant recipients who were transplanted in collaboration with TLC. The information that is available is sparse, making analysis of statistical trends impossible, and is a direct reflection of the challenging, resource-limited environments in which partner centres are operating. Data are available for 16 paediatric patients who have been transplanted, seven females and nine males (Table 1). Where known, causes of kidney failure include the following:

- Obstructive uropathy
- Bilateral vesicoureteral reflux
- Neurogenic bladder
- Systemic lupus erythematosus (SLE)
- Chronic glomerulonephritis of unknown aetiology
- Congenital kidney dysplasia

**Table 1:** Paediatric kidney transplants performed with TLC assistance

Patient number	Sex	Pre-transplant KRT	Diagnosis	Age at transplant	Present graft status^a^	Present age
Centre 1						
1	M	HD	Obstructive uropathy	16	Failed—back on HD	19
Centre 2						
2	M	PD	Chronic glomerulonephritis of unknown aetiology	17	Deceased	
3	M	HD	Bilateral vesicoureteric reflux	17	Functional—eGFR 52	22
4	F	PD	Congenital kidney dysplasia	12	Functional—eGFR 95	16
Centre 3						
5	F	HD	Unknown	17	Failed—back on HD	
6	F	HD	Spina bifida/neurogenic bladder	14	Failed—back on HD	
7	M	HD	Nephrotic syndrome	11	Awaiting HD—eGFR 16	26
8	F	HD	Unknown	16	Failed—back on HD	29
9	M	PD	Reflux nephropathy	10	Deceased	
10	M	PD	Reflux nephropathy	11	Failed—back on HD	23
11	F	HD	SLE	16	Failed—back on HD	
12	M	HD	Unknown	15	Functional—eGFR 87	24
13	F	HD	Unknown	14	Functional—eGFR 112	22
14	M	HD	Immune complex nephritis (IF showed IgG and C3 mesangial and peripheral positivity)	12	Deceased	
15	M	PD	Reflux nephropathy	14	Functional—eGFR 89	14
16	F	None	Reflux nephropathy	12	Functional—eGFR 116	13

^a^eGFR values given in ml/min/1.73 m^2^

Patients received either peritoneal dialysis or haemodialysis via a central venous catheter before transplantation. Donors were most commonly one of the child’s parents, but two brothers and an uncle of patients have also acted as donors. In all cases, hand-assisted laparoscopic donor nephrectomies were performed uneventfully; all donors made a full recovery with outcomes comparable to those seen in high-income countries. Transplants were performed in all 16 patients, with no graft loss or postoperative mortality in the first year.

In total, seven patients have returned to dialysis post-transplantation. The time to return to dialysis ranged from 2 years and 5 months to 13 years and 3 months post-transplantation. Biopsies were performed for a subset of these patients, and findings included cellular rejection, chronic allograft nephropathy, and BK virus nephropathy. For those patients with currently functioning grafts, the latest available eGFR values range from 16 to 116 ml/min/1.73 m^2^.

Three patients have died following transplantation: one patient developed a lymphoma post-transplantation and died secondary to complications from this, one patient died in a road traffic accident, and the cause of death of the final patient is not known.

Completion of these paediatric transplants by partner centres is a significant achievement; however, TLC recognises that patient outcomes are notably poorer than those of paediatric transplant recipients in high-income countries [19]. Many challenges were faced in the performance of these transplants, several of which paralleled those seen in well-established transplant programmes in high-income countries. Ensuring post-transplant compliance with immunosuppression regimens and fluid balance monitoring has been difficult, particularly for teenage patients on the verge of transitioning to adult services. There have also been significant psychosocial and emotional challenges. Transplantation is often viewed as a cure, so patients and their families have found it difficult to accept complications when they arise, particularly the need to restart dialysis following graft failure. This highlights the damage that can occur when the expectations of patients and families do not match the realities of transplantation and the importance of comprehensive work-up and counselling pre- and post-transplantation. Families also often face ongoing financial challenges, particularly in being able to afford the ongoing costs of medications where they are not covered by the health service and the impact of the burden of this falls on the whole family.

In addition to these widely recognised challenges, many further factors have additionally complicated and contributed to worse outcomes for the paediatric transplants performed in partnership with TLC. Medications routinely used in the management of CKD in high-income countries were inconsistently available at partner centres. Although data were not formally collected by TLC, it was noted by the medical faculty that most paediatric patients had advanced CKD-associated complications such as poor nutrition, hypertension, mineral bone disease, and anaemia prior to being transplanted. In high-income countries where CKD in children is aggressively managed, patients have much better baseline health and rarely develop such severe comorbidities before transplantation. Thus, given their often suboptimal CKD management and resultant comorbidities, transplant outcomes of paediatric patients at partner centres were never going to be comparable to those seen in high-income countries. Nonetheless, transplantation offered these patients an opportunity to improve their life expectancy compared to continuing on dialysis. Moreover, access to standard post-transplantation protocol medications as well as specialist therapies and interventions for complications is much more limited in LMICs, making it more challenging to provide the same quality of care and follow-up that patients receive in high-income countries. As a result, post-transplant complications that would be successfully managed and treated in high-income countries are associated with much higher morbidity and mortality in LMICs.

## Summary of TLC paediatric kidney transplantation symposia

In addition to supporting the training of a paediatric nephrologist with a placement in the UK, TLC has delivered monthly online symposia for over 2 years, attended by people across 113 different countries. Within these symposia, aspects of paediatric kidney transplantation are specifically covered (Table 2). These symposia are live-streamed to virtual audiences, enabling real-time engagement and interaction with the presenter, with recordings subsequently available via the TLC website for viewing by other healthcare professionals unable to attend the live events.

**Table 2:** TLC symposia on paediatric kidney transplantation

Symposium topic	Number of registrations	Number of countries represented amongst registrations	Maximum number of attendees observed during live symposium
Preparing a paediatric patient for transplant: psychological considerations	161	22	91
Challenges in paediatric kidney transplantation in a new transplant centre	86	24	49
Challenges in managing the post-transplant paediatric recipient	113	32	46
Managing transition from the paediatric to the adult service	95	29	48

## Challenges experienced by TLC

Over the past 15 years, TLC has helped establish and develop paediatric kidney transplant services across multiple LMICs. The charity’s work demonstrates that potential barriers to transplantation can be overcome and that, with the correct combination of tuition, assistance, and mentorship, partner centres can work toward having self-sufficient transplant programmes. However, despite individual successes, the paediatric transplant services supported by TLC have encountered many challenges (summarised in Table 3) that have contributed to the observed poor outcomes for transplanted children in partner centres. These areas remain key priorities in the charity’s ongoing work but are issues for which quick and easy solutions are rarely available, further emphasising the importance of TLC’s philosophy of a longstanding commitment to transplant programme development.

**Table 3:** Challenges in setting up paediatric kidney transplant services experienced by TLC

Initial challenges	Long-term challenges
• Local government funding and will to develop services • Identifying local teams with paediatric nephrology, urology, and kidney transplantation expertise • Sub-optimal management of CKD • Identifying healthy donors • Reliable supplies of medication and surgical equipment • Causes of kidney failure often unknown	• Lack of paediatric nephrology medical, nursing, and allied professional clinicians • Turnover of key staff • Variable level of clinical care • Inconsistent political support • Variable availability of immunosuppression and monitoring • Social issues, clinic non-attendance, and non-concordance with medication • Infrastructure for clinical data collection

Despite significant developmental progress having been made, it is disappointing that this has invariably been slower than expected and that progress has consistently been punctuated by periods of inactivity. These observations are not a reflection of the capability or enthusiasm of the clinicians involved. On the contrary, our experience has been that with appropriate support provided by the TLC model, they rapidly acquire the skills required, adapting them to local circumstances to develop and maintain high-quality local programmes. However, these local champions are frequently constrained by local challenges that are beyond their control. In short, adequate and consistent funding may be absent; while political support may initially be given to the development of programmes, this may not translate into the resources required to develop and sustain a comprehensive paediatric nephrology service. As such, while development may occur with full mentoring support, the point of self-sustainability may continue to be out of reach. Accordingly, based on the TLC experience, it seems evident that in addition to providing practical assistance, the international transplant community must support clinicians in LMICs in lobbying their governments to provide the additional resources required to support the needs of children with kidney failure, particularly the full development of kidney transplant programmes.

## Conclusion

A huge disparity remains in paediatric nephrology care provided worldwide, with the availability and access to dialysis and kidney transplantation being particularly sparse in LMICs. As such, CKD continues to cause significant excess paediatric morbidity and mortality in LMICs. Further global action is needed to ensure the next generation of paediatric patients with kidney failure has access to the required kidney transplants. Transplantation professionals, as well as policymakers, must take responsibility to act as advocates for children currently dying from kidney failure, a readily treatable disease, and lobby for change. The progress of TLC partner centres demonstrates that LMICs can aspire to their own transplant programmes. Also, TLC’s model is effective when there is a long-term commitment from all parties involved to overcome the significant hurdles that currently threaten development. The involvement of TLC in transplant programmes in the Caribbean has led to the recognition that the challenges identified in this paper apply to other LMICs in which the charity has worked. TLC hopes that its activity can catalyse further progress; however, making paediatric kidney transplantation a reality for children with kidney failure globally will not be possible without the necessary political vision and financial resources.

## Data Availability

All data generated or analysed during this study are included in this published article.
